# The metabolic associated fatty liver disease responses of lifestyle changes using diet and exercise

**DOI:** 10.12669/pjms.39.6.7990

**Published:** 2023

**Authors:** Benash Altaf, Mahaneem Mohamed, Shireen Jawed, Wan Syaheedah Wan Ghazali

**Affiliations:** 1 Benash Altaf, PhD Assistant Professor, Physiology, Aziz Fatimah Medical and Dental College, Faisalabad Punjab, Pakistan; 2 Mahaneem Mohamed Associate Professor, Physiology, Universiti Sains Malaysia, Malaysia; 3 Shireen Jawed Associate Professor, Physiology, Aziz Fatimah Medical and Dental College, Faisalabad Punjab, Pakistan; 4 Wan Syaheedah Wan Ghazali Lecturer, Physiology, Universiti Sains Malaysia, Malaysia

**Keywords:** Metabolic associated fatty liver, Quality of life, Adult, Lifestyle modification

## Abstract

**Objective:**

This review is aimed to study MAFLD responses of lifestyle modifications using Diet and Exercise.

**Methods:**

The sources for this MAFLD review following PRISMA protocol were PubMed, Google scholar, Scopus and Science Direct. Quality of evidence was assessed by consistent results with previous studies. Assessment of quality was done by Joanna Briggs Institute criteria. Quality of evidence was assessed by GRADE approach tool.

**Results:**

This review included 12 studies, from which five were qualitative and seven quantitative. The later showed poor dietary habits and sedentary lifestyle exhibiting MAFLD which eventually affect their quality of life. Further studies suggested that by introducing healthy lifestyle in MAFLD group using diet and exercise caused reduction in BMI, obesity levels, improved glycemic control and reversal of liver fat content with improved liver enzymes.

**Conclusion:**

Subjects with MAFLD experienced poor quality of life. Altering lifestyle by diet and exercise can improve their physical wellbeing.

## INTRODUCTION

 Metabolic associated fatty liver disease (MAFLD) is a condition which is increasing at the alarming rate worldwide. It is worth worrying that its prevalence is currently reported 25% globally which indicates that it is affecting quarter of our adult population worldwide.^1^,^2^ On the other hand, solely considering Asia, it has increased to 40% from 2012-2017, thence has increased the economic load and has made it a global challenge, as becoming the leading cause for liver transplant also.^3^ Formerly, MAFLD was known as non-alcoholic fatty liver disease (NAFLD) which is now replaced to new nomenclature MAFLD as this covers broad range of its associated metabolic features.

 This increasing trend of diabetes and obesity which are part of metabolic features are responsible for increasing burden of MAFLD.^3^ Researchers suggests that the timely focus on lifestyle modification and dietary habit might play a crucial role for the effective management of this condition.^4^ MAFLD criteria is based on hepatic steatosis on ultrasonography along with presence of one of the following three criteria i.e. increased weight/obesity, Type-2 diabetes and presence of metabolic dysregulation including interrelated biochemical abnormalities dyslipidemia and other factors like central obesity, and hypertension. While Metabolic dysregulation is defined as the presence of two or more of these conditions which include Blood pressure, increases waist circumference, Triglycerides, HDL < 40 mg/dl, and C-reactive protein (CRP) levels >2 mg/L.^5^,^6^

 It is evident that in certain group of people having higher risk of disease progression. Different epidemiological studies document that its pathophysiology is closely inter-related to obesity, increased adipose tissue stores, dyslipidemia, insulin resistance and diabetes,^7^,^8^ thereby it is related to unhealthy dietary habits, and lack of physical activity, reflecting role of environmental risk factors for its pathogenesis.^8^,^9^ Diet has a profound effect as well as considered as potent risk factor for causing NAFLD with its associated metabolic characteristics leading it towards MAFLD, specifically, a diet rich in fructose and fats play a crucial role.^10^ It is shown that through energy restriction and dietary macronutrients alteration, including restriction of carbohydrates, fat, or enriched monounsaturated fatty acids, can be the factors responsible for reducing metabolic syndrome.

 Moreover, Westernized dietary pattern, that include red meat, refined grains, bakery items, and sugar based beverages are linked with a greater likelihood for the development of metabolic syndrome and subsequent MAFLD.^11^ Sedentary lifestyle with poor physical activity as well as long sitting or lying hours is well proved to be culprit for obesity and increased lipid content in respective individuals. This poor lifestyle lacking physical activity leads to increased BMI, body fat and circulating lipid content causing fatty soft tissue organs, hypertension, insulin resistance, diabetes leading to metabolic syndrome.^12^,^13^

 Currently, despite much work on MAFLD and metabolic associated features yet no definite therapeutic consensus are established for its management and treatment. For this reason, this review aimed to evaluate the relationship of quality of life (QOL) and Metabolic Associated Fatty Liver Diseases - (MAFLD), and how lifestyle change using diet and exercise regimen could have causative impact on treating patients with MAFLD.

## METHODS

### Protocol and registration

are registered on International Prospective Register of systematic Reviews PROSPERO (CRD42022310685). Our systematic review was reported following guideline of Preferred Reporting Items for Systematic Reviews and Meta-Analyses (PRISMA) for assuring its transparency.

### Inclusion and exclusion criteria:

All full text original /Research studies, prospective retrospective and randomized control trial written in English assessing QOL in adult patients with MAFLD, using validated instruments were included in the current review. We included only human studies in which the terms NAFLD and MAFLD were used alternatively. After going through the details, only MAFLD studies with quality of life were included. We excluded pediatric MAFLD/NAFLD/NASH as it needed separate review. We also excluded NAFLD studies on thin lean individuals without having metabolic dysregulation thence, not fulfilling the MAFLD criteria. We also did not consider studies in which methodology was not on QOL which was explicitly defined and measured according to the World Health Organization. We used a data collection report to extract data for each study, including first author name, study year, place of study, number and demographic data of participants, method of MAFLD diagnosis, tools used to assess QOL and major outcomes. We also excluded studies in which QOL was not taken as a primary outcome of MAFLD.

### Search and Selection:

All data was extracted using MeSH and non-MeSH terms on various search engines including PubMed, SCOPUS, Google scholar and Science Direct for query “Impact of lifestyle modifications on quality of life of adult metabolic associated liver disease (MAFLD) patients”. All data related to lifestyle modification AND quality of life OR QOL OR Well-being AND MAFLD OR metabolic associated Fatty Liver Disease OR NAFLD AND adults were extracted from 2005 till 2022. The search terms were reformed according to the search criteria for every database. Articles were searched using following search terms in the following combination: Lifestyle modification AND quality of life OR QOL OR well-being AND MAFLD OR metabolic associated fatty liver disease OR NAFLD AND adults.

 Our search focused exclusively on English full text papers that encompassed a detailed description of effect of MAFLD on quality of life. BA and SJ screened all titles and abstracts that were retrieved following the inclusion criteria. After that, all reviewers discussed each article’s eligibility in order to reach a final consensus at rate of almost 90%. Articles that had conflicting opinion, concerning the inclusion for articles was resolved by senior co-author.

 Information related scores of QOL among MAFLD and Control with p-values, impact of how lifestyle modification improves QOL, general characteristics of the study related to methodology was also extracted, saved, and reviewed. Duplicate articles were removed with reference manager software (Mendeley). Papers for title, abstract, and full text were screened by two independent authors separately following the already designed set of rules. In the case of any disagreement, a consensus was reached after discussion with a third author. Full text research with cohort or case-control, cross sectional and surveys were considered eligible. The score of QOL with different tools are presented with Euro QOL 5D, HRQL4, and SF36.

### Data Extraction:

Two reviewers BA and SJ performed data extraction from eligible studies into a standardized data collection Form. A third independent author resolved disagreements. The following information was extracted from each study: first author, year of publication, digital object identifier, study design, study period, the number of centers, study site (country), the number of patients, the number of participants with and without MAFLD, associated factors with MAFLD like obesity, dyslipidemia diabetes, and mean and SD of QOL scores, odd ratio (OR) if available and p values were reported. All the data extracted was maintained on the separate Excel sheet to minimize the error.

### Risk of Bias and quality assessment:

Risk of biasness was maximally avoided by adopting two independent review authors who carried out the review. Conflicts were resolved by taking help of third reviewer. Critical assessment Joanna Briggs institute (JBI) criteria was used for quality of the review which was done with help of checklist in order to make the review more validated.^14^

## RESULTS

### Search and Selection:

Research questionnaire: “Does lifestyle modification in metabolic associated fatty liver disease (MAFLD improves their quality of life and wellbeing” was registered on PROSPERO, utilizing the MeSH key words. Total 659 articles were retrieved from PubMed (n=92), Google Scholar (n=445) SCOPUS (n=11) and Science Direct (n=41) from 2015 till 2022. After sorting the duplicate articles (n=143), reviews and meta-analysis (n=67), articles on pediatric studies (n=51) and irrelevant (n=343), 55 research articles were further dealt for the title and abstract. Out of these, 33 research papers did not fulfilling the MAFLD criteria, paid articles and full text articles which were not available were further excluded.

**Fig.1:**
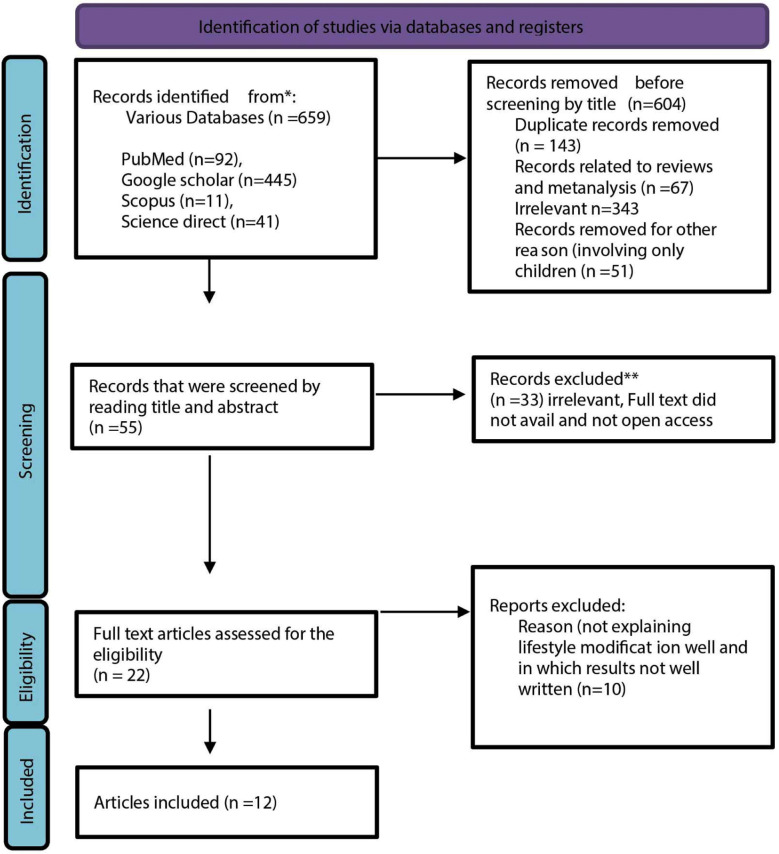
Selection of papers for review.

 The remaining 22 full text articles were thoroughly studied and the 10 research articles were further excluded not meeting the criteria of lifestyle modification as some articles only considered lifestyle modification as altering only dietary habits while other considered only adopting increase physical activity in the form of exercise. Thereby only 12 studies were analyzed meeting the complete eligibility criteria (Fig.1).

### Characteristics of the studies:

Characteristics of the studies are shown in Table-I.

**Table-I:** Characteristics of the studies included in the systemic review of MAFLD and non- MAFLD subjects

Study Author	Study Year	Country	Studied subject	Associated factor	Study Design	Sample size	Tool used
Tea in Hwang 2021	2010-12	Korea	NAFLD	hyperlipidemia, hypertension, insulin resistance	Survey	17476	Euro QOL 5D
Golabi etal^15^ 2016	2001-11	America	NAFLD	Increased BMI hyperlipidemia, hypertension	Survey	9661	HRQOL-4
Rui-Huang^16^ 2021	Mar-Aug 2019	China	NAFLD	obesity	Survey	5181	CLDQ-NAFLD
Kristin David^17^ 2009	2002	America	NAFLD	diabetes	Survey	713	SF36
Niharika Samala^18^ 2020	2017-19	Indiana University	NAFLD	Diabetes mellitus, hypertension, Obesity	Survey	341	SF36

### Quantitative synthesis:

Quantitative synthesis for comparing QOL in MAFLD and non-MAFLD is shown in Table-II.

**Table-II:** Quantitative synthesis comparing quality of life score among MAFLD and non- MAFLD subjects.

Study Author	Study Year	Scale	Effect size	Model	Quality score MAFLD	Quality Score non- MAFLD	P value
Tea in wang^19^	2010-12	Euro QOL 5D	OR	Logistic regression	0.95±0.001	0.94±0.004	0.018
Golabi etal^15^	2001-11	HRQOL-4	OR	Logistic Regression	The overall HRQOL score was lower (mean±SD not given)	Over HRQOL were comparatively Good	<0.0001
Rui-Huang^16^	Mar-Aug2019	CLDQ-NAFLD	OR	Logistic Regression	5.66 ± 0.8	NA	NA
Kristin David^17^	2002	SF36	OR	Regression analysis	mean PCS score 55.8, MCS score 52.5)	NA	<0.0001
Niharika Samala^18^	2017-19	SF36	NA	Pearson correlation	Physical component summary score-43.9±10.21 General health-50.9322.±46	Not Given	<0.0001

### Qualitative synthesis:

Qualitative synthesis was done for 12 RCTs. RCT conducted by Abdelbaset et al, 2020 observed 47 NAFLD diabetic obese thereby fulfilling MAFLD criteria. Subjects were divided into three groups, including home walking, and stretching exercise (control), moderate aerobic intensity exercise and high intensity Ergometer exercise with 16 subjects in each group with one drop out from moderate intensity exercise group. Exercise was under guidance of physiotherapist. Pre and post hepatic fat content (HFC) as well as base line were recorded. Dietary modification detail was not mentioned by the author. Statistically significant similar reductions in HFC, BMI, blood sugar levels and liver enzymes were noted following moderate and high intensity exercises showing both exercises had same beneficial effect in MAFLD subjects but not in the control group. In addition, both of exercise programs i.e., high intensity exercise, 80% to 85% HRmax, and moderate intensity exercise, 60% to 70% HRmax both for three sessions/week for eight weeks showed significant decreases in hepatic lipid and TGs content. The authors reported that the results were also supported by previous studies conducted by various researchers (Table-III).^20^

**Table-III:** Qualitative synthesis for impact of intervention on quality of life in MAFLD and control healthy individuals.

Author with year	Study Type	Population	Duration	Intervention	protocol for intervention	protocol for intervention for control	changes after intervention	Assessment	P-value
Abdelbasset et al2020^20^	RCT	47 NAFLD diabetic	8weeks	exercise and diet	Dietary control, high intensity and low intensity exercise	only home walking and stretching exercise	changes after intervention reduced BMI, reduction in blood sugar level and liver enzyme	Paired t test	<0.05
Katsagoni et al ,2018^21^	RCT	NAFLD & obese(BMI greater than 31	24weeks	Both exercise and diet	Energy restriction by CHO, protein & lipid with 60 min small sessions to how improve diet every month. Also guided them for taking rest as well as a small power nap at day along with guidance on physical activity	dietary guidelines were provided in written for energy restriction for CHO, protein and lipid	decreased weight and BMI, with less liver stiffness and improved liver enzymes and triglyceride TAG	ANOVA and Regression	<0.05
Arab et al,2017^22^	RCT	81 subject with Fatty liver on USG and raised liver enzymes and obesity	9weeks	Both exercise and diet	eight small session on guiding healthy eating and regular physical activity	simple care	decreased weight and BMI with decreased abdominal circumference and lean body mass	test of co variance	<0.05
Author with year	Study Type	Population	Duration	Intervention	protocol for intervention	protocol for intervention for control	changes after intervention	Assessment	P-value
Cheng et al, 2017^23^	RCT	115 recruited NFALD with diabetes obesity and altered liver enzymes 85 completed follow up	37 weeks	exercise and diet	aerobic exercise were guided along with dietary plan	No measurement	Decreased hepatic fat content	NA	P value for hepatic fat-0.006 P value for Liver enzymes-NS
Dong et al.2016^24^	RCT	280 fatty liver on USG (141 caee,139 control)with BMI greater than25	104 weeks	exercise and diet	Lifestyle counseling	No Intervention	decreased weight, and BMI and triglyceride, and improved NAFLD scoring	NA	P value BMI-0.03 ALT- P<0.01 AST P value NS
Eckard et al, 2013^25^	RCT	56 obese NAFLD subjects diagnosed on liver biopsy w	26 weeks	exercise and diet	Exercise logs and pedometer use were discussed weekly by an exercise physiologist to monitor compliance.	Standard care	decreased BMI and improved fatty liver status and improved liver enzymes	ANOVA (pre post diff)and Pearson correlation with variables	significantly different outcome
Al-Jiffri O et al,2013^26^	RCT	100 historic diagnosed NAFLD subjects with BMI 30-35 and diabetes 2	13weeks	exercise and diet	Low caloric diet was advised endurance training session for 15 minutes	no intervention	decreased BMI and improved fatty liver status and liver enzymes	T test to compare pre and post lifestyle modification among 2 groups	significantly different outcome

 Katsagoni et al, 2018 observed obese NAFLD following the lifestyle modification and its impact on QOL, dividing subjects into three groups (Control, Mediterranean diet, and lifestyle groups) with 21 subjects per group. Mediterranean diet group was on Mediterranean diet comprised of plants and grain only. Control group was directed to follow healthy lifestyle as according to guidelines provided without any intervention (Table-III). Compliance to Mediterranean diet and Mediterranean lifestyle that comprised of self-employed moderate-vigorous physical activity program of at least 30 minutes/day, was checked by physician supervisor every two weeks. Subjects were again divided into three groups, first group was following the diet, and second group was following the exercise and third group as control having equal 21 participants in all. But unfortunately, seven dropped-out from the control group. Mediterranean food or Mediterranean lifestyle, both are found to be helpful in improving liver enzyme, status of liver texture and weight reduction. However, no significant reduction of these parameters in control group was noted. Moreover, there was a significant difference among the control group and the groups following Mediterranean diet and lifestyle.^21^

 Arab et al in 2017 also observed lifestyle modification on 82 subjects in fatty liver obese subjects who had altered liver enzymes, thence fulfilling the MAFLD criteria. Participants were guided properly to utilize more fruits and vegetable, complex carbohydrates, fish, and diet low in fat dairy, with cut down of unhealthy fat and refined carbohydrates. In addition, one session was arranged for physical activity with regular 30-60 minutes exercise for five days/week. They observed significant difference in outcomes in the form of reduction of all aspects of MAFLD that comprised it as compared to control, in which following diet and exercise, subjects experienced decreased weight, BMI, abdominal circumference and lean body mass.^22^

 Similar study performed by Chang et al, 2017 recruited 115 fatty liver subjects with both obesity and diabetes. Out of these, 30 subjects dropped out. They made four groups i.e., exercise, diet, both exercise and diet, and no intervention groups. This study found that diet alone as well as the group solely on exercise had significant reductions of HBA1c, serum AST and ALT, and BMI in MAFLD subjects as compared to no intervention group. They also found that subjects who followed aerobic exercise and diet showed markedly significant reduction in HFC after 37 weeks of intervention.^23^

 Dong et al, 2016 encouraged subjects using the diet having 23~30% fat (1/3 saturated and 2/3 unsaturated fatty acids), 15~20% protein, and 50~60% carbohydrate, and for physical activity they mostly targeted the activities, that increases heart rate greater than 80 percent, like heavy jogging, swimming, cycling and aerobics for 4/weeks and for 60/session. For accuracy assurance, all processes were supervised. Before intervention, no baseline difference was found between control and test groups, but after intervention, they observed significant reductions in abdominal circumference, BMI and liver enzymes in test group compared to control group. Al-Jiffri O et al, 2013 divided subjects into two groups i.e., dietary modification with exercise. They reported improved parameters of MAFLD including liver enzyme and BMI, moreover, improvement was more significant among the subjects receiving dietary modification with exercise. They also reported significant difference in outcomes among control and MAFLD subjects (Table-III).^24^-^26^

### Quality of evidence:

Quality of evidence was assessed by the consistent results of the previous studies and publication biased and limitation of their methodology. As the included studies adopted the proper protocol for methodology and selection criteria for their enrollment of participants, there is little chance of bias. Results of included studies are also supporting and in-line with the previous literature, so there is no inconsistency. The enrolled population of those studies is desired population and match with the goal of the current reviews (no-indirectness). Based on these parameters of GRADE approach indicates that evidence of this current review is moderate to high.

## DISCUSSION

 Prevalence of MAFLD is increasing at alarming rate due to sedentary lifestyle and poor dietary habits.^27^ This systemic review focuses on impact of lifestyle modification on quality of life of adult MAFLD subjects. In this review, Hwang & Han (2021) reported poor QOL using tool Euro QOL 5d in MAFLD diabetics in Korean population as compared to the controls. They reported significant odds ratio, indicating MAFLD subjects are 1.077 times more at risk of poor QOL as compared to non-MAFLD subjects.^19^ Golabi et al, conducted a survey on American population utilizing the tool HRQOL-4, reporting that MAFLD subjects were associated with other comorbidities including hyperlipidemia (57.01%), hypertension (58.99%), and insulin resistance (66.3%). They also reported significantly poor score among MAFLD subjects compared to non-MAFLD subjects.^15^

 Another study conducted by Huang et al in China with CLDQ-NAFLD form observed that obese NAFLD subjects have poor QOL.^16^ David et al on the other hand also agreed and reported the similar statistical significant difference for poor QOL among MAFLD and non-MAFLD group utilizing the tool SF36.^17^ Samala et al conducted a study utilizing SF36 form, recruiting subjects from American Indiana University. Their recruited NAFLD subjects were associated with hypertension, diabetes, and obesity. Almost 85% of study subjects were obese, 48% with diabetes while 55% with hypertension, thereby making it as MAFLD subjects. They reported that these MAFLD subjects though not owing advanced fibrosis, exhibited significantly poor QOL than control group.^18^

 A study conducted in Cairo observed the effects of dietary modifications and exercise on QOL of NAFLD diabetic subjects, termed as MAFLD, documented significant improvement in quality of life of these patients following the dietary control and exercise. This study reported significant reduction in BMI reflecting the reduction in body lipid content, with improvement in blood sugar levels and liver enzymes following the moderate intensity and high intensity aerobics exercises. These findings suggested that both the high and moderate intensities exercises along with the dietary control are beneficial for NAFLD diabetic patients (MAFLD)^20^ which are in line with the previous study.^28^

 A study conducted in Greece by Katsagoni et al, also documented reduction in body weight and regularization of liver enzymes following the Mediterranean diet and moderate intensity physical activity in NAFLD patients.^21^ Supporting to aforementioned study, previous Iranian study in 2017 observed lifestyle modification in obese NAFLD subjects, reporting significant reduction in body weight and BMI after guided diet control with regular exercises but they did not observe significant difference for the liver enzymes. Hence, this study suggested that dietary control along with physical activity improves the QOL of these patients.^22^ Similar study was conducted by Chang et al in 2017 in Shanghai, China on NAFLD obese and diabetic patients. This study is slightly different and unique from above studies as this study had observed the effect of diet control or exercise alone as well as in combination of both dietary and exercise for 37 weeks on the obese diabetic NAFLD patients (MAFLD), also making it a longer duration than all other above-mentioned studies.

 This study reported improvement in BMI, liver enzymes and HBA1c, and reduction in HFC in all the study groups, however the greatest reduction in HFC was observed in the group following combined strategy.^23^ This study results are also in line with the previous research conducted by Gomez showing significant reduction in HFC as well as liver biomarkers and blood glucose levels.^29^ Similar results has been documented by Dong et al, Eckard et al and Al-Jiffri O et al studies conducted at China and King Abdul Aziz University, reported significant difference in outcomes among control and MAFLD subjects, with significantly decreased BMI, triglycerides and liver enzymes, and improved HFC status.^24^-^26^ Collective findings of all studies reviewed in current systematic review suggested that dietary control and physical activities have beneficial effects in decreasing weight, liver enzymes and HFC of MAFLD patients, thus improving quality of life of these patients.

## CONCLUSION

 MAFLD subjects experience poor quality of life. Lifestyle modification including diet modification and exercise can improve the physical wellbeing ultimately improving the general health which will eventually help to lessen the severe outcomes and burden of disease on our society.

### Author’s Contribution:

**BA** and **SJ:** Selected the topic for current review, performed literature search and, data extraction, manuscript writing, quality assessment and risk of bias. Registered this review with PROSPERO.

**MM:** Involved in topic selection, guide for all the protocols of PRISMA for literature search and PROSPERO registration. Review all included articles for inclusion criteria and quality assessment and risk of bias. Reviewed all intellectual contents of systematic review and made all required corrections.

**WSWG**: Helped in topic selection, checked all the included articles for inclusion criteria, quality assessment and risk of bias, and helped in manuscript revision. All authors are considered for accountability of data.
